# Genome Wide Association Study for Predictors of Progression Free Survival in Patients on Capecitabine, Oxaliplatin, Bevacizumab and Cetuximab in First-Line Therapy of Metastatic Colorectal Cancer

**DOI:** 10.1371/journal.pone.0131091

**Published:** 2015-07-29

**Authors:** Jan Pander, Lieke van Huis-Tanja, Stefan Böhringer, Tahar van der Straaten, Hans Gelderblom, Cornelis Punt, Henk-Jan Guchelaar

**Affiliations:** 1 Department of Clinical Pharmacy & Toxicology, Leiden University Medical Center, The Netherlands; PO box 9600, 2300 RC Leiden, The Netherlands; 2 Department of Clinical Oncology, Leiden University Medical Center, The Netherlands; PO box 9600, 2300 RC Leiden, The Netherlands; 3 Department of Biostatistics and Bioinformatics, Leiden University Medical Center, Leiden, The Netherlands; 4 Department of Medical Oncology, Academic Medical Center Amsterdam, The Netherlands; PO Box 22660, 1100 DD Amsterdam, The Netherlands; Medical University of Graz, AUSTRIA

## Abstract

**Purpose:**

Despite expanding options for systemic treatment, survival for metastatic colorectal cancer (mCRC) remains limited and individual response is difficult to predict. In search of pre-treatment predictors, pharmacogenetic research has mainly used a candidate gene approach. Genome wide association (GWA) studies offer the benefit of simultaneously analyzing a large number of SNPs, in both known and still unidentified functional regions. Using a GWA approach, we searched for genetic markers affecting progression free survival (PFS) in mCRC patients treated with first-line capecitabine, oxaliplatin and bevacizumab (CAPOX-B), with or without cetuximab.

**Patients and Methods:**

755 patients were included in the CAIRO2-trial, a multicenter phase III trial, randomizing between first-line treatment with CAPOX-B versus CAPOX-B plus cetuximab. Germline DNA and complete clinical information was available from 553 patients and genome wide genotyping was performed, using Illumina’s OmniExpress beadchip arrays, with 647,550 markers passing all quality checks. Another 2,202,473 markers were imputated by using HapMap2. Association with PFS was analysed using a Cox proportional hazards model.

**Results:**

One marker, rs885036, associated significantly with PFS (P = 2.17x10^-8^) showing opposite effects on PFS depending on treatment arm. The minor allele was associated with increased PFS in patients receiving cetuximab. A cluster of markers located on chromosome 8 associated with PFS, irrespective of treatment arm (P-values of 2.30x10^-7^ to 1.04x10^-6^).

**Conclusion:**

This is the first GWA study to find SNPs affecting PFS in mCRC patients treated with CAPOX-B, either with or without cetuximab. Rs885036 is a potential predictive marker for cetuximab efficacy. These markers need to be validated in independent treatment cohorts.

## Background

Colorectal cancer is among the most prevalent forms of cancer worldwide, with the estimated number of new diagnoses in the United States for 2013 exceeding 142,000 cases.[1] Combination chemotherapy including a fluoropyrimidine plus the monoclonal agent bevacizumab and either oxaliplatin or irinotecan is generally recommended for first-line treatment of metastatic colorectal carcinoma (mCRC).[2] Unfortunately, even with these modern-day treatment options, median survival is limited and variation in response is largely unpredictable. Optimal selection of patients who will benefit from these extensive treatment schedules is warranted, both from the individual aspect of preventing needless burden of toxicity, and from a population-based aspect of optimal cost-effectiveness.

Germline genetic variation has been shown to predict differences in response to many chemotherapeutic drugs.[3] Until now, most research has focused on genetic alterations in genes encoding for known target or metabolic enzymes. A disadvantage of this candidate-gene approach is the limited knowledge of the exact mechanism of action for many drugs. Considering the immense amount of single nucleotide polymorphisms (SNPs) the human genome harbors, it is likely that many SNPs with potential effect on drug efficacy have not yet been detected. Circumventing the limitations of a candidate gene approach, genome wide association (GWA) studies offer the possibility of simultaneously analyzing a large number of SNPs, even in regions that have not previously been associated with the drug under investigation. This type of study has been applied to identify risk factors for multiple types of cancers, including colorectal carcinoma.[4,5] Moreover, in pharmacogenetic GWA studies, an increasing number of SNPs associated with treatment response in cancer are identified. A recent GWA study in colorectal cancer patients identified SNPs that were associated with adverse drug reactions in response to treatment with 5-FU and oxaliplatin.[6]

Here, we present the results of the first clinical GWA study to find SNPs that are associated with progression free survival (PFS) of first-line combination chemotherapy for mCRC with capecitabine, oxaliplatin and bevacizumab (CAPOX-B), either with or without cetuximab.

## Patients and Methods

Germline DNA was obtained from 553 of 755 previously untreated mCRC patients, who were recruited from the CAIRO2 trial, a multicenter phase III trial of the Dutch Colorectal Cancer Group (DCCG), which randomized between first-line treatment with CAPOX-B versus CAPOX-B plus cetuximab.[7] All patients received three-weekly cycles of capecitabine 1000mg/m^2^ b.i.d. orally on days 1–14 plus oxaliplatin 130mg/m^2^ (maximum of 6 cycles) and bevacizumab 7.5mg/m2, both intravenously on day 1 of each treatment cycle. From cycle 7 the capecitabine dosage was increased to 1250mg/m^2^. For patients randomized to treatment with CAPOX-B plus cetuximab, cetuximab was administered intravenously at a loading dose of 400mg/m^2^ on the first treatment day, followed by 250mg/m^2^ once weekly thereafter. Treatment was continued until disease progression, unacceptable toxicity or death, whichever occurred first. Patient eligibility criteria and guidelines for response assessment have been described in detail elsewhere.[7] The study was approved by the Committee on Research involving Human Subjects Arnhem-Nijmegen and by all local institutional ethics boards. All subjects gave written informed consent.

### Genotyping

Whole blood was collected at baseline and germline DNA was isolated from peripheral leukocytes using MagnaPure Compact (Roche diagnostics, Almere, The Netherlands). Genotyping was performed on Human OmniExpress v12 BeadChip arrays containing 733,202 markers (Illumina, San Diego, CA, USA). Genotype calls were set using GenomeStudio software (Illumina). All products were used according to manufacturer’s prescriptions. The following cut-off values were used to filter out incorrectly called genotypes: GenCall ≥ 0.85; ClusterSep ≥ 0.3; CallFreq >0.85; AB T-mean 0.2–0.8, resulting in the exclusion of 3172 markers (0.43%).

### Statistical analysis

#### Quality control

Quality control was performed using R, version 3.0.1 (http://www.r-project.org/), and *plink*.[8] Markers were excluded based on a minor allele frequency (MAF) threshold of 0.01 (1636 excluded markers; 0.3%) and missingness threshold of 0.05 (no excluded markers). Hardy-Weinberg equilibrium (HWE) was evaluated per marker, using a χ^2^ goodness-of-fit statistic with a cut-off P-value of ≤10^−7^ (36 excluded markers). After these quality checks, 647,550 markers remained in the analysis.

Individuals were excluded based on missingness > 2% (no excluded individuals).

**Fig 1:**
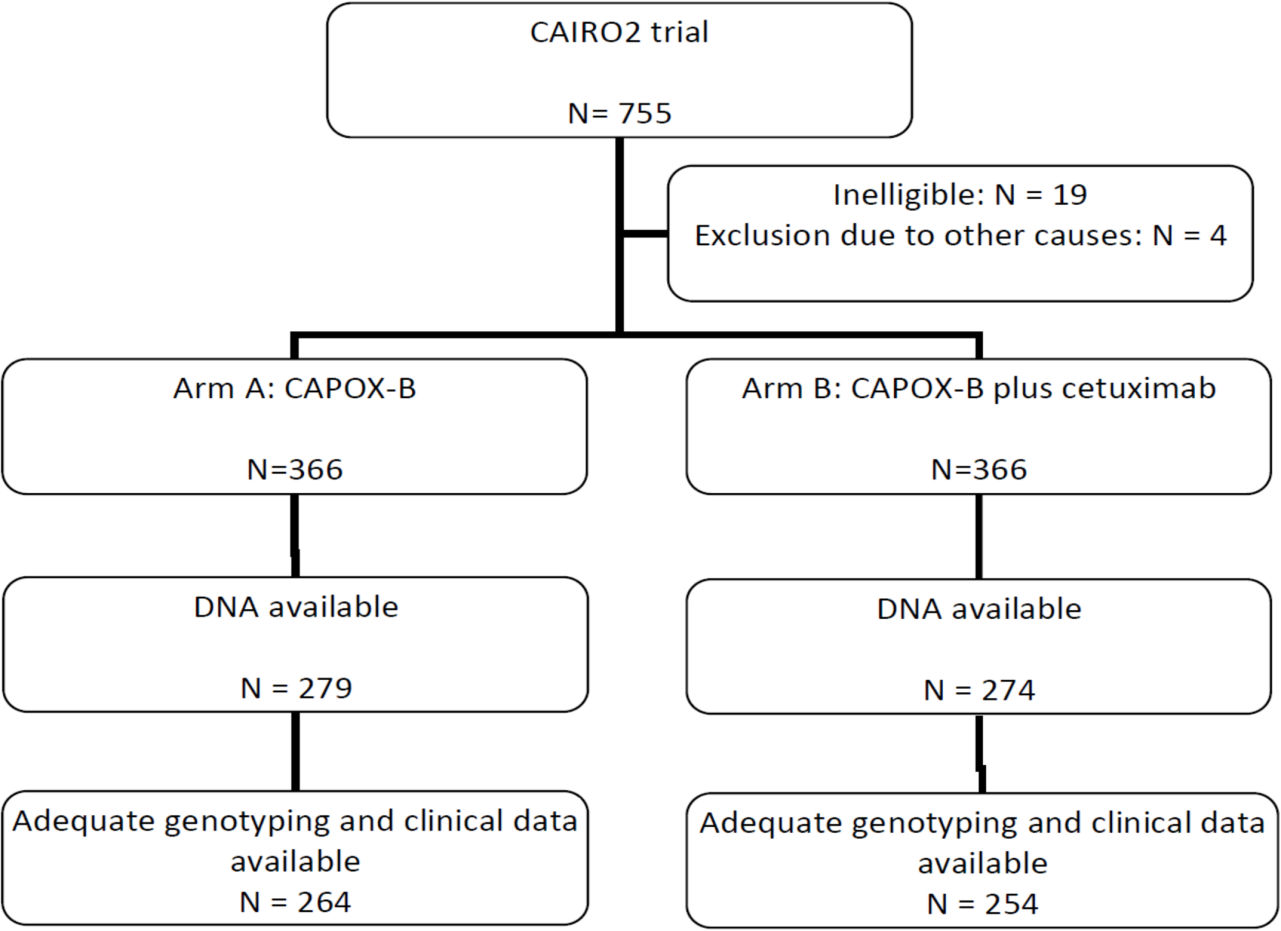
Cairo2 study flow-chart.

Multidimensional scaling (MDS) was used to investigate possible stratification and outliers. After removal of outliers MDS did not show clustering. Association analysis was performed with and without the first four MDS coordinates as covariates. Ranking and P-values of the top 30 SNPs were almost identical and only analyses without MDS coordinates are shown. In total 33 patients were excluded due to low genotyping quality. Complete information, including genotype, was available for 520 patients. The study flowchart is shown in Fig 1.

#### Imputation

Additionally, non-measured genotypes were imputated using HapMap2, to further enhance SNP density. A minimum imputation accuracy of R^2^>0.40 was applied to select reliably imputed SNPs. In total 2,202,473 imputed markers were used in this analysis.

#### Association model

For each marker, the association with PFS was calculated with a Cox-proportional hazards model using *R* package *survival*. Age, gender, treatment arm were included as covariates. A first model contained each SNP as variable of interest (marginal model) and a second model tested the interaction SNP*arm as variable of interest while controlling for the main SNP effect (interaction model). Markers were evaluated using an additive genetic model in all analyses. The inflation factor for association models was calculated based on the χ^2^-quantiles for the P-values of the evaluated models. Formal significance for a marker was assumed for a two-sided P < 5x10^-8^ to correct for multiple testing, as has been described by others.[9–11] Kaplan-Meier curves were estimated for the marker with the lowest P-value using *R*.

## Results

### Base-line characteristics

Baseline patient characteristics are described in Table 1 (online only). Median PFS was 10.3 months for all patients included in the analyses (range 0.1–44.7 months). At the time of analysis the primary endpoint PFS was reached by 487 of 520 patients (93.7%).

**Table 1:** Baseline patient characteristics.

	All patients	CAPOX-B	CAPOX-B plus cetuximab
	(n = 520)	(n = 264)	(n = 256)
**Age in years**			
Median (range)	63.2 (27.6–83.6)	62.9 (27.6–83.6)	63.9 (33.2–80.0)
**Sex–no (%)**			
Male	316 (60.8)	144 (54.5)	172 (67.2)
Female	204 (39.2)	120 (45.5)	84 (32.8)
**LDH at baseline–no (%)** ^1^			
Normal	307 (59.0)	152 (57.6)	155 (60.5)
Above upper limit of normal	213 (41.0)	112 (42.4)	101 (39.5)
**Previous adjuvant chemotherapy–no (%)**			
Yes	443 (85.2)	223 (84.5)	220 (86.6)
No	71 (13.7)	37 (14.0)	34 (13.4)
Unknown	6 (1.2)	4 (1.5)	2 (0.8)
**Progression free survival**			
Median (range)	10.3 (0.1–44.7)	10.6 (0.4–44.7)	10.0 (0.1–40.8)

^1^ According to cut-off values of each individual center

CAPOX-B, capecitabine, oxaliplatin and bevacizumab; LDH, lactate dehydrogenase.

### Genome-wide association analysis including effect of treatment arm

**Fig 2:**
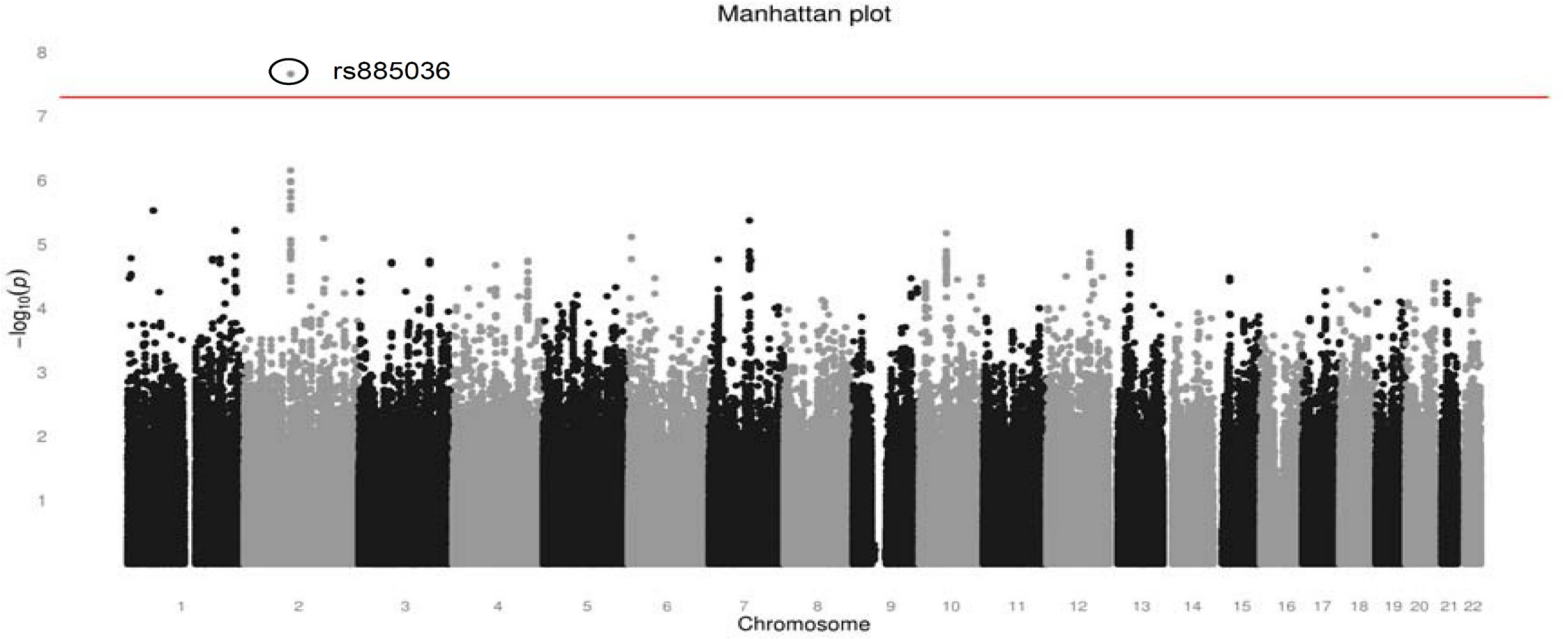
Manhattan plot of–log_10_ (P-value) of the Cox-proportional hazards model. Adjusted for age, gender and treatment arm and the interaction of marker with treatment arm. The horizontal line represents the formal genome-wide significance level of 5x10^-8^.

**Fig 3:**
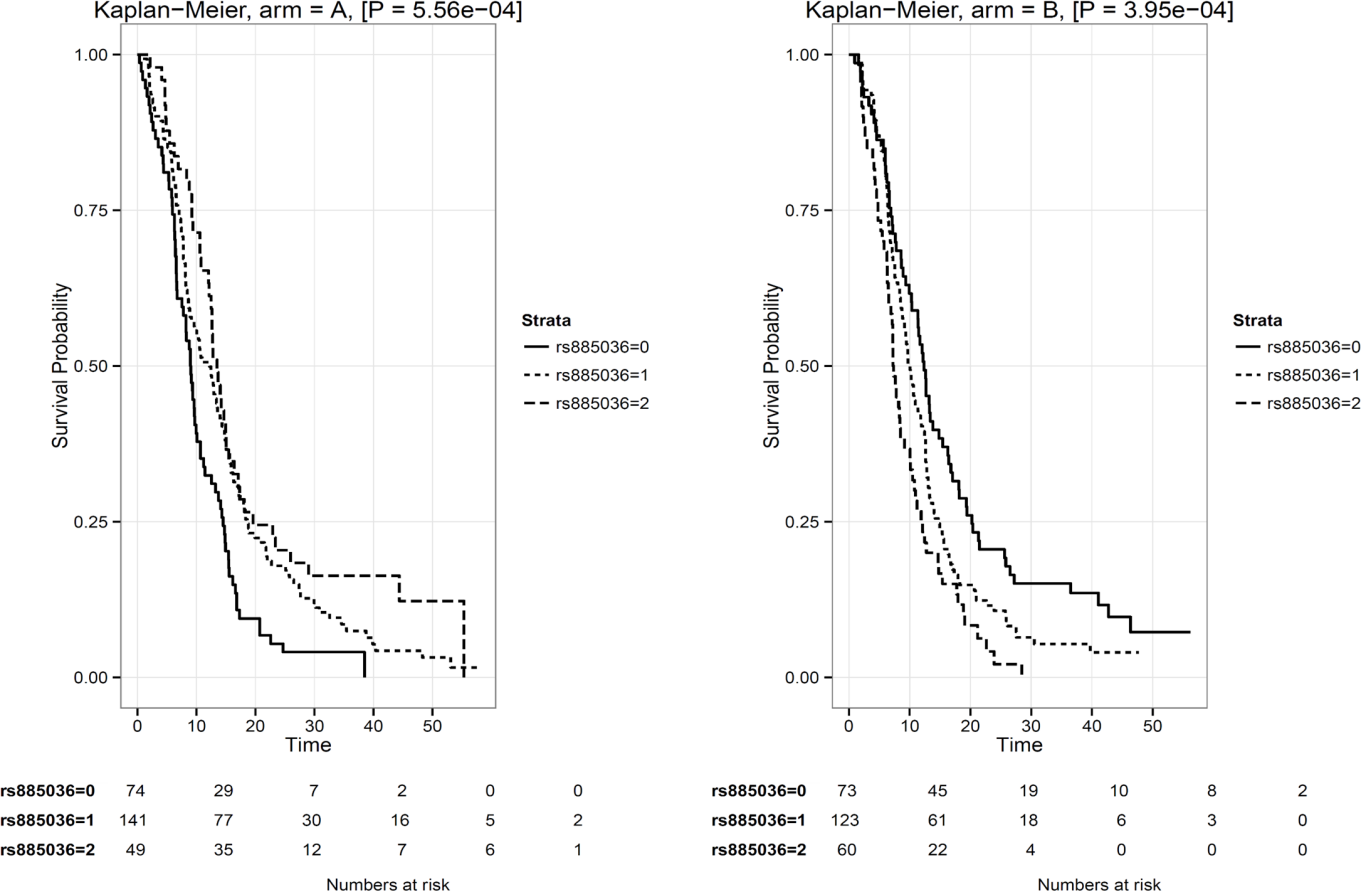
Kaplan-Meyer survival curves according to rs885036 genotype. A. Survival curves for patients in arm A, treated with CAPOX-B in first-line chemotherapy. B. Survival curves for patients in arm B, treated with CAPOX-B plus cetuximab in first-line chemotherapy.

Initially, analyses were performed including the interaction term of genetic markers with treatment arm as a covariate. The Manhattan plot for these analyses is shown in Fig 2 and details for the ten most significant markers are provided in Table 2. Markers with a differential effect according to treatment arm may actually reflect predictive markers for cetuximab efficacy. One marker (rs885036, position 98671226) on chromosome 2q12 showed a highly significant interaction with treatment arm (P = 2.17x10^-8^). In a stratified analysis, this SNP proved to have a contrasting effect in both treatment arms. Kaplan-Meyer survival curves for PFS according to genotype are shown in Fig 3. For patients treated with CAPOX-B, the G allele associated with shorter PFS (AA 13.47 months [95%CI 12.16–16.43]; AG 12.22 months [95%CI 9.17–14.32]; GG 9.00 months [95%CI 7.56–10.68]). For patients treated with CAPOX-B plus cetuximab, the G allele associated with increased PFS (AA 7.31 months [95%CI 6.53–10.05]; AG 10.05 months [95%CI 8.83–12.16]; GG 12.35 months [95%CI 10.35–15.44]).

**Table 2:** Ten SNPs with lowest P-values for association with PFS.

Marker	Chr	Position	Gene	A0	A1	Allele freq	R^2^	Allele HR* (95% CI)	P-value
rs885036	2	98671226	*GnT- IVa*	A	G	0.533	1.000	0.47 (0.36–0.61)	2.17x10^-8^
rs3769689	2	98660129	*GnT- IVa*	A	G	0.327	0.975	0.49 (0.37–0.65)	6.99x10^-7^
rs3769688	2	98660242	*GnT- IVa*	A	G	0.329	0.980	0.50 (0.38–0.66)	1.02x10^-6^
rs17448420	2	98664315	*GnT- IVa*	C	T	0.329	0.983	0.50 (0.38–0.66)	1.06x10^-6^
rs17448190	2	98656831	*GnT- IVa*	C	T	0.325	0.972	0.50 (0.38–0.66)	1.06x10^-6^
rs17448211	2	98657628	*GnT- IVa*	A	T	0.325	0.973	0.50 (0.38–0.66)	1.07x10^-6^
rs17448085	2	98653118	*GnT- IVa*	C	T	0.328	0.974	0.50 (0.38–0.67)	1.49x10^-6^
rs17514013	2	98653226	*GnT- IVa*	A	G	0.672	0.974	1.99 (1.50–2.64)	1.49x10^-6^
rs10199926	2	98658908	*GnT- IVa*	C	T	0.656	0.982	1.96 (1.49–2.60)	1.87x10^-6^
rs2309434	2	98672936	*GnT- IVa*	C	T	0.340	0.987	0.51 (0.39–0.68)	2.46x10^-6^

Abbreviations: HR, Hazard ratio; Chr, chromosome; CI, confidence interval; GnT-IVa: mannosyl (alpha-1,3)-glycoprotein beta-1,4-N-acetylglucosaminyltransferase isozyme A; R2: imputation accuracy. A0: reference allele according to hapmap2, A1: alternate allele.

* HR assuming an additive effect depending on dosage of the alternate alleles, obtained with multivariate Cox-proportionale hazards model, including age, gender, treatment arm, and the interaction treatment arm* marker as covariates.

### Overall genome-wide association analysis results

**Fig 4:**
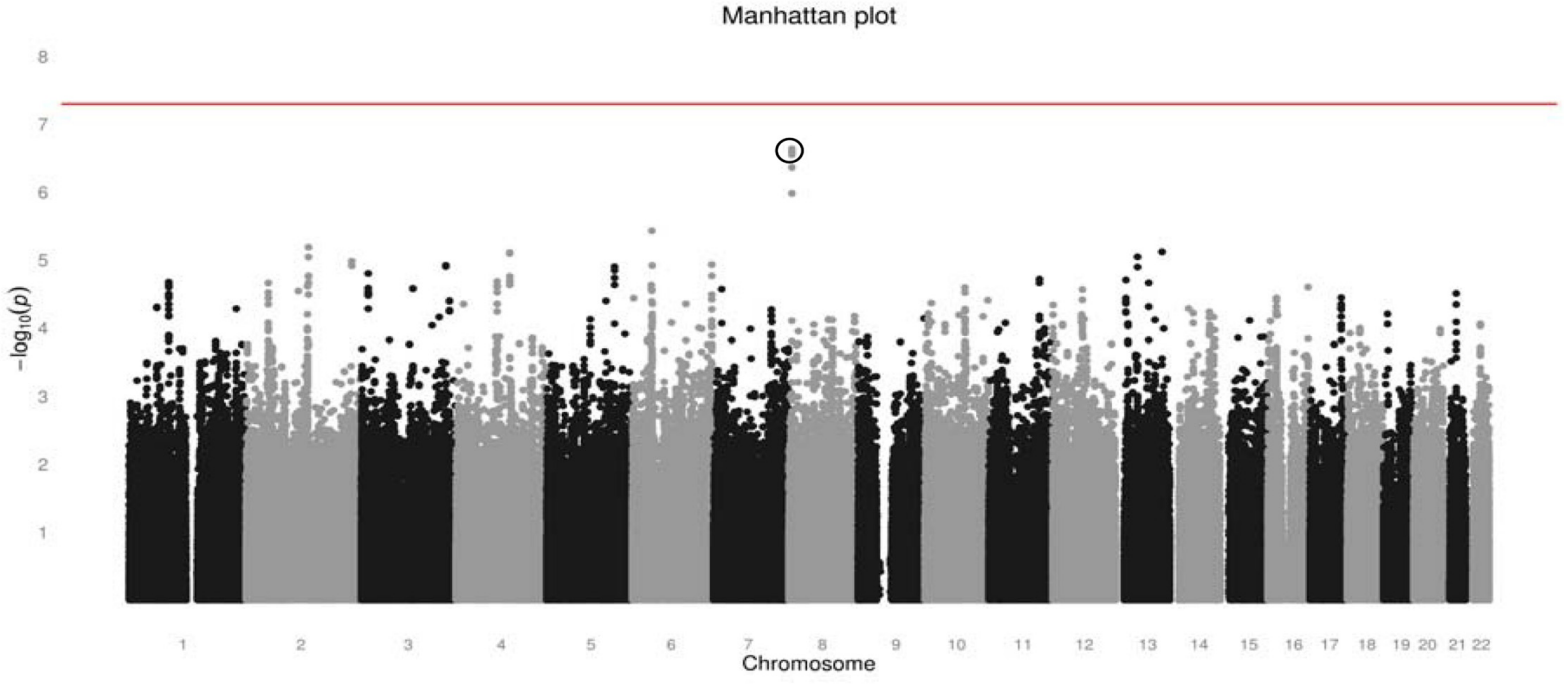
Manhattan plot of–log_10_ (P-value) of the Cox-proprotional hazards model. **Adjusted for age, gender and treatment arm.** The horizontal line represents the formal genome-wide significance level of 5x10^-8^. The topmost significant markers are circled, i.e. rs2936519, rs2928608, rs2928609, rs2912024, rs2978926, rs2928607.

The Manhattan plot for the overall GWA analysis is shown in Fig 4. No marker reached the pre-determined formal significance level. A cluster of markers, located on chromosome 8, showed the lowest P-values (P = 2.30x10^-7^ to P = 1.04x10^-6^, respectively). The inflation factor for this analysis was modest (λ 1.02), indicating there was no evidence of population stratification or other bias in the analysis. The ten most significant markers are described in Table 3.

**Table 3:** Ten SNPs with lowest P-values for overall association with PFS.

Marker	Chr	Position	Gene	A0	A1	Allele freq	R^2^ *	Allele HR^#^ (95% CI)	P-value
rs2936519	8	6626650	*n*.*a*.	A	G	0.90	1.00	0.53 (0.43–0.67)	2.30x10^-7^
rs2928608	8	6626434	*n*.*a*.	C	T	0.90	0.99	0.54 (0.43–0.67)	2.33x10^-7^
rs2928609	8	6626528	*n*.*a*.	C	T	0.90	0.99	0.54 (0.43–0.67)	2.36x10^-7^
rs2912024	8	6626309	*n*.*a*.	C	T	0.10	1.00	1.86 (1.49–2.32)	2.60x10^-7^
rs2978926	8	6626835	*n*.*a*.	A	G	0.10	0.98	1.86 (1.49–2.33)	2.67x10^-7^
rs2928607	8	6626120	*n*.*a*.	C	G	0.10	0.97	1.86 (1.49–2.33)	2.79x10^-7^
rs11997869	8	6625874	*n*.*a*.	C	G	0.10	0.97	1.84 (1.47–2.30)	4.32x10^-7^
rs2978931	8	6625491	*n*.*a*.	A	C	0.91	1.00	0.55 (0.44–0.69)	1.04x10^-6^
rs2073016	6	41128900	*n*.*a*.	C	T	0.83	0.72	1.62 (1.31–2.00)	3.70x10^-6^
rs4377367	2	131441375	*ARHGEF4*	C	T	0.86	0.99	0.63 (0.52–0.76)	6.47x10^-6^

Abbreviations: HR, Hazard ratio; Chr, chromosome; CI, confidence interval; *n*.*a*., marker is not localized within a gene. ARHGEF4: Rho guanine nucleotide exchange factor 4.

* R2: imputation accuracy. A0: reference allele according to hapmap2, A1: alternate allele.

^#^ HR assuming an additive effect depending on dosage of the alternate alleles, obtained with multivariate Cox-proportionale hazards model, including age, gender, treatment arm, and the interaction treatment arm* marker as covariates.

## Discussion

This is the first prospective GWA study to find SNPs that predict efficacy of first-line chemotherapy with CAPOX-B, with or without cetuximab, in mCRC patients in a clinical trial setting. One marker on chromosome 2, rs885036, was significantly associated with PFS, with a contrasting effect in both treatment arms. This SNP may therefore be a potential predictive marker for efficacy of cetuximab containing chemotherapy. Additionally, a cluster of SNPs on chromosome 8 influenced PFS with a similar effect in both treatment arms, almost reaching genome wide significance.

A genome-wide significant effect on PFS was found for rs885036, when treatment arm was taken into consideration. Interestingly, we found opposite effects of genotype on PFS in both treatment arms. This suggests that this polymorphism may have a deleterious prognostic effect on survival per se, but this effect is no longer seen after the addition of cetuximab to standard treatment with CAPOX-B.

Rs885036 is located in the gene encoding for mannosyl (alpha-1,3)-glycoprotein beta-1,4-N-acetylglucosaminyltransferase isozyme A, *GnT-IVa* (also denominated MGAT4A), as are all ten most significant markers from these analyses. The top region of most significant markers exhibits long ranging linkage disequilibrium (LD), extending >50kb in both directions (HapMap3 data, data not shown), which also covers the candidate gene *GnT-IVa*. Of note, rs885036 is a directly measured SNP.

*GnT-IV* encodes for a glycosyltransferase, which is involved in the biosynthesis of oligosaccharides and has previously been associated with tumor behavior. In one study, *GnT-IV* mRNA was upregulated in metastatic colorectal tumors, as opposed to non-metastatic primary colorectal tumors.[12] Strong expression of *GnT-IVa* mRNA and enzyme activity was also found in drug-resistant hepatocellular carcinoma cells.[13]

The rs885036 SNP constitutes a C>T nucleotide change in the second intron of *GnT-IVa*, and is located within a predicted binding site for the highly conserved microRNA-34A (miR-34a).[14,15] MicroRNAs play an important role in post-transcriptional silencing of genes, as their interference with RNA leads to repressed translation or cleavage of RNA.[16] Expression of miR-34a itself is regulated by p53 expression.[17] Cytotoxic stress induced by chemotherapy or irradiation markedly increases miR-34a levels in the presence of p53[18], emphasizing its importance in response to anti-cancer treatment.

We believe that the C>T change leads to altered binding of miR-34a, resulting in differential translation efficacy of *GnT-IVa*. Although our analyses cannot clarify any further functional correlations, we speculate that the altered GnT-IVa protein level may then influence EGFR glycosylation, and thereby form a possible determinant of cetuximab efficacy.

Although no SNP in the overall analyses reached formal genome wide significance, a cluster of SNPs on chromosome 8p23.1 showed very low P-values for the association with PFS. Within the cluster, are two directly genotyped SPNs, including the most significant SNP (rs2936519).

These marers are not localized within any known gene, but the gene encoding for 1-acylglycerol-3-phosphate O-acyltransferase 5 (*APGAT5*), is found approximately 20 kbp upstream.[19] Again, the topmost significant SNPs are in long ranging LD, which covers this candidate gene (HapMap3 data, data not shown). AGPAT5 comes from a family of highly conserved enzymes that catalyze the acylation of lysophosphatidic acid (LPA) to phosphatidic acid, the second step in the *de novo* formation of glycerophospholipids.[20,21] Although the function of most AGPAT isoforms is largely unknown, multiple AGPAT isoforms have been linked to cancer. AGPAT9 and 11 are overexpressed in colon cancer tissue, as well as in other cancer types.[20,22] AGPAT2 inhibition was shown to induce *in vitro* growth arrest and cell death in different tumor types, including several colon cancer cell lines.[23]

To our knowledge, this is the first prospective GWA study investigating systemic treatment efficacy in Caucasian mCRC patients. Only two previous similar studies have been published worldwide, and none of the markers identified by those experiments overlap with our results.[24,25] These studies, both by the same Korean research group, followed a three step design, validating results from GWA analysis in both an independent patient cohort and transfected colorectal cancer cell lines. They found two SNPs with putative influence on efficacy of cetuximab-containing regimens for colorectal carcinoma[25], as well as one SNP influencing disease recurrence after adjuvant chemotherapy with 5-FU for stage II or III colorectal carcinoma.[24] In both studies, the selection of markers to be carried forward to the validation studies was based on the minor allele frequency and location in a haplotype block in the Asian population, and only non-synonymous SNPs were included. Considerable differences in allele frequencies exist between Asian and European populations for the relevant genetic markers from both our and Kim’s analyses, which in part may explain the lack of overlap between our results.[19] Also, considerably larger treatment heterogeneity was present in their population, thereby possibly diluting the effect of germline genetic markers on treatment efficacy.

Our study is subject to several limitations. Interpretation of GWA studies in general is hampered by the need for large patient populations, to ensure sufficient power to detect genotype effects. With 547 patients, our cohort used for this GWA study is larger than many other pharmacogenetic study populations. Non-significant results could therefore lead to the conclusion that indeed none of the tested SNPs are associated with PFS upon CAPOX-B treatment in mCRC. However, since the power to detect associations depends not only on population size, but also on the size of a SNP-effect and on genotype frequency. Despite a relatively large sample size, false negative results could arise for SNPs with low minor allele frequencies. In fact, minor allele frequencies of the three topmost significant SNPs from the overall analyses are only 0.10 in our population, which may well have led the results to be non-significant, despite an actual effect on PFS.

Obviously, it can be argued that rare variants or markers with a small effect on treatment efficacy are not clinically relevant, irrespective of their statistical significance level. However, genetic predisposition is unlikely to depend on only a few SNPs for most drug effects. Rather, multiple genetic markers could attribute in small amount and interact with each other.[26,27] Their combined effect, in conjunction with clinical and pathologic parameters, could be of value in predictive models. Such a model has recently been proposed for colorectal cancer risk assessment.[28] In future, similar models should be constructed for predicting treatment response as well as adverse events for diverse systemic treatment regimens. In this scenario, any SNP influencing the complex trait ‘drug efficacy’ is of value.

Another drawback of GWA analyses is that these studies only focus on the influence of genetic variation, without taking into consideration most patient and tumor characteristics. Whereas in the time the CAIRO2 trial was performed cetuximab was prescribed to unselected patients, regardless of tumor characteristics, it was shown shortly thereafter that patients carrying a somatic mutation in *Kras* codon 12 or 13 are resistant to treatment with cetuximab.[29–32] More recently, patients with mutations in *Nras* were also shown not to benefit from treatment with anti-epidermal growth factor antibodies.[33] More somatic mutations precluding EGFR inhibitor efficacy may be discovered.

At the time the study was conducted, EGFR inhibitor therapy was not restricted to Kras wild type tumors only. However, *Kras* mutation status has been analyzed restrospectively in those patients for whom tumor DNA was available. For this subgroup, we performed additional analyses, taking *Kras* codon 12 and codon 13 alleles into consideration. We found that rs885036 remained an independent predictor for PFS, with only a small change in HR (HR 0.466 without *Kras*, HR 0.472 with *Kras* as a covariate; Table 4).

**Table 4:** Ten SNPs with lowest P-values for association with PFS.

Marker	Chr	Position	A0	Allele frequency	Allele HR*	P-value
Rs8180209	4	90863476	G	0.08	0.24	2.83x10^-01^
Rs530376	5	152819508	G	0.45	0.46	5.06x10^-01^
Rs11830274	12	90597138	A	0.02	0.03	7.28x10^-01^
Rs11837647	12	90624226	A	0.02	0.03	7.28x10^-01^
**Rs885036**	**2**	**98671226**	**A**	**0.46**	**2.12**	6.87x10^-01^
Rs3118146	1	180533882	C	0.23	2.32	2.95x10^-01^
Rs2477185	1	180533140	A	0.23	2.32	3.19x10^-01^
Rs1776148	1	240109167	A	0.36	2.14	9.27x10^-02^
Rs1025159	1	163741907	A	0.15	0.37	7.67x10^-01^
Rs3025363	9	135471888	A	0.38	0.47	5.87x10^-01^

Abbreviations: HR, Hazard ratio; Chr, chromosome; A0: reference allele according to hapmap2

* HR assuming an additive effect depending on dosage of the alternate alleles, obtained with multivariate Cox-proportionale hazards model, including age, gender, treatment arm, *Kras* mutation status and the interaction treatment arm* marker as covariates.

Recently, it was found that patient survival in colorectal cancer is influenced by different molecular subtypes, including not only *Kras* mutation status, but several other genetic and epigenetic factors.[34] Because molecular subtype was not determined in the CAIRO2 population, we cannot completely exclude an effect of molecular subtype on the outcome of our analyses. However, there is no evidence in literature for an association between *GnT-IVa* genotype and colorectal cancer molecular subtype.

Contrary to practice in clinical trials, serum LDH level at baseline, although strongly correlated to PFS[7], was not included in our multivariate model. Confounding by the prognostic effect of LDH cannot be excluded, since there was a slight overrepresentation of patients with increased LDH levels in several genotype groups with shorter PFS (data not shown). LDH level in itself may be a predictive as well as a prognostic factor, and not completely independent of germline genotype. Serum LDH-levels have been associated with intratumoral gene expression of vascular endothelial growth factor type A (*VEGFA*) and vascular endothelial growth factor receptor type 1 (*VEGFR1*).[35] Both phenomena are thought to be the result of stimulation of hypoxia-inducible factor 1α (HIF-1α), due to the intratumoral aerobic glycolysis known as the Warburg effect.[36] Results of a recent study suggest that, although high serum LDH levels correlate with reduced survival in colorectal cancer patients, treatment with bevacizumab can improve PFS for patients with high pre-treatment LDH levels to that of patients with normal baseline LDH levels.[37] Because an interaction of LDH with genotype cannot be excluded, and inclusion of LDH in the experimental model could possibly have obscured genotype effects, we chose not to correct for the effect of LDH level at baseline in this study.

## Conclusion

In this GWA study, investigating the effect of genetic variation on PFS on first line CAPOX-B with or without cetuximab in mCRC, we found one marker with a significant effect on treatment efficacy, with opposing results in each treatment arm.

This SNP, rs885036, may be of predictive importance for patients treated with cetuximab-containing regimens, and should be further validated in patients with RAS wildtype tumors. Patients in our sample carrying the rs885036 GG genotype seem to respond better to CAPOX-B plus cetuximab, whereas patients with the AA genotype benefit more from CAPOX-B alone. Therefore, it is possible a subgroup of patients carrying the GG genotype may benefit from the addition of cetuximab to CAPOX-B, contrary to overall findings for the general study population of CAIRO2 and study with comparable design.[7,38] Whether this marker also predicts PFS in patients treated with cetuximab monotherapy should be subject to further research. We formulated a hypothesis for the pathophysiological basis of this effect, which we are now testing in pre-clinical studies.

In addition, a cluster of SNPs on chromosome 8 with very low P-values was found, which may also have functional significance. For these SNPs, we cannot differentiate between a prognostic effect of these polymorphisms on PFS and a predictive effect in relation to chemotherapy. These results should also be validated in other populations.
